# Is leisure beneficial for older Korean immigrants? An interpretative phenomenological analysis

**DOI:** 10.3402/qhw.v11.33103

**Published:** 2016-11-30

**Authors:** Junhyoung Kim, Sangjeong Moon, Jungsun Song

**Affiliations:** 1 Central Michigan University, Mt Pleasant, MI, USA; 2 School of Airline Tourism, Gumi, South Korea, Republic of Korea; 3 International Tourism Management, Daegu, Republic of Korea

**Keywords:** Coping, leisure, interpretative phenomenological analysis, psychological wellbeing, social support

## Abstract

Leisure is an important quality of life factor for older Korean immigrants. The purpose of this study was to explore leisure benefits associated with health among older Korean immigrants. A total of 18 individuals participated in the study. Using interpretative phenomenological analysis (IPA), three themes emerged from participants’ personal statements and experiences: (a) experiencing psychological benefits, (b) strengthening social connections, and (c) coping with acculturative stress. The findings indicate that leisure provided a context in which older Korean immigrants created an emotional and social support system that helped them to experience psychological and social benefits. This research suggested that older Korean immigrants used leisure as a coping mechanism that results in health and well-being.

Leisure is one of the important discussions among older Asian immigrants. The meaning of “leisure” is defined as “a fun, entertaining activity that people engage in during their free time.” Leisure scholars suggest that participation in leisure can help older Asian immigrants to facilitate an adaptation process and improve health and well-being. A growing body of literature has provided evidence that leisure contributes to physical, social, and mental health among older adults (Hamer, Molloy, De Oliveira, & Demakakos, 2009; Lampinen, Heikkinen, & Ruoppila, 2000). For example, Kim, Yamada, Heo, and Han (2014) found that participation in community-based leisure activity clubs, such as gateball, dance, and table tennis, facilitated positive psychological feelings, encouraged positive social relationship, and enhanced their physical functions.

Furthermore, research indicates that leisure served as effective coping resources and strategies for older adults (Bagheri-Nesami, Raftii, & Oskouie, 2010; Fitzpatrick et al., 2008). These authors found that by participating in various activities such as gardening, cultural activity, and home-based activities, older adults developed the ability to cope well with their stressors and enhanced their health and well-being. As evidence, Demers, Robichaud, Gélinas, Noreau, and Desrosiers (2008) found that older adults used social activity participation, such as social gatherings, as a coping strategy and, as a result, they reduced the effect of stressful life events associated with age.

Despite the apparent benefits of leisure engagement, there is a research gap with regard to leisure behaviors associated with acculturation and immigration experiences among older Asian immigrants. Among a diversity of older Asian group immigrants, in this study a group of older Korean immigrants was chosen for the following reasons: First, older Korean immigrants are the fourth largest Asian immigrant group in the United States; however, there is limited research about this population (Berkman & Ko, 2009). Furthermore, compared to other older Asian immigrants, older Korean immigrants seem to face more challenges regarding their adaptation and experience lower levels of English skills and acculturation (Jang & Chiriboga, 2010; Lee & Yoon, 2011). It can be argued that older Korean immigrants tend to experience acculturative stress and negative psychological symptoms. Therefore, based on gaps within the current literature that focus on older Korean immigrants’ leisure behaviors related to acculturation, this study aimed at exploring leisure benefits associated with health among older Korean immigrants. Three questions were used to guide the present study:

1. What do older Korean immigrants do for fun or entertainment in their free time (i.e., leisure activities)?
2. Why do older Korean immigrants participate in leisure activities?
3. What benefits do older Korean immigrants experience through leisure participation during the immigration process?

## Adaptation challenges experienced by older Asian immigrants

The term “acculturation” is defined as a “… process of cultural and psychological change that follows intercultural contact” (Berry, Phinney, Sam, & Vedder, 2006, p. 305). Researchers suggested that acculturation, particularly from non-Western cultures to Western cultures, is a stressful event (Blair, 2012; Mori, 2000; Park & Rubin, 2012). The major challenges that older Asian immigrants experience are a lack of social support from the host society, family conflicts, financial hardships, racial discrimination experiences, and difficulty using health care services (Chung & Epstein, 2014; Huang, Nam, & Lee, 2014). These impacts are usually greater for older immigrants compared to younger immigrants because the older immigrants experience their aging processes as well as acculturation to new cultures (Jang, Roh, & Chiriboga, 2014). Specifically, the different values of the older immigrants between their original culture and a new culture (e.g., collectivism vs. individualism, interdependence vs. independence) might cause high acculturative stress (Lee & Yoon, 2011; Yamamoto, Rhee, & Chang, 1994).

Furthermore, older Korean immigrants identified language barriers, isolation/loneliness, fear of being a burden on their children, financial issues, transportation problems, discrimination, and fear of death as acculturative stressors, which are related to their adjustment and socioeconomic changes because of immigration (Lee & Yoon, 2011). Mui and Kang (2006) found that these cultural gaps and acculturative stressors were related to depression among older Asian immigrants.

## Leisure benefits among older Asian immigrants

A body of leisure literature shows evidence that leisure engagement has been associated with positive outcomes, such as decreased levels of depression and stress (Chang, 2014; Fernández-Fernández, Márquez-González, Losada-Baltar, & Romero-Moreno, 2014), acculturation-related personal growth (Kim & Kim, 2013), and increased health perception (Chang, Yu, & Jeng, 2014). The primary contributor to these positive outcomes can be the ability of leisure to provide rich opportunities for older adults to socialize with others, ameliorate negative psychological symptoms, and increase physical ability.

More directly, numerous studies have reported the positive relationships between physical leisure activities and physical well-being (e.g., Adams, Leibbrandt, & Moon, 2011; Cuenca, Kleiber, Monteagudo, Linde, & Jaumot-Pascual, 2014). Furthermore, Dionigi (2006) indicated that the older persons’ participation in exercises increased their self-confidence toward their life and body as well as future health condition. It also improved their functional ability and psychological well-being (Bath & Morgan, 1998; Laukkanen, Kauppinen, & Heikkinen, 1998).

In a recent study, Kim, Lee, Chun, Heo, and Han (2014) observed the effects of engaging in leisure-time physical activity on psychological benefits, such as optimism, positive affect, psychological well-being, and life satisfaction, for older adult immigrants; they found that participation in leisure-time physical activity was positively associated with all of these. Through leisure-time physical activity involvement, the older adult immigrants attained optimism, improved their positive affect, and increased their psychological well-being and life satisfaction. Such benefits can lead to greater quality of life and successful aging.

## Methods

Previous studies indicated that a qualitative approach seems to be the most appropriate to explore lived experiences and health-related benefits (e.g., Kim, Heo, & Kim, 2014; Kim, Kim, Han, & Chin, 2015). For this study, interpretative phenomenological analysis (IPA) was used for detailed structural analysis of the interviews, as it aims to explore meanings of particular experiences (Smith, Flowers, & Larkin, 2009). This analysis is most suited to studies that aim to explore participants’ experiences in their interactions with the environment (Smith, Jarman, & Osborn, 1999). This adheres to the purpose of this study, which is to explore older Korean immigrants’ leisure benefits.

### Participants

Purposeful criterion sampling strategy was applied to this study as suggested by Patton (2002), as it helps researchers to have valid interpretation of each case and understand certain social phenomenon of participants’ life experiences. The criteria for the participants were as follows: (a) they had legally moved to the United States and were Korean American citizens and/or had permanent residence; (b) they could speak, read, and understand Korean or English; and (c) they were aged 65 years or older (this is a figure based on comparisons with previous studies). A total of 18 individuals participated (10 men and 8 women), with age range 65–85 years (*M*=72.2). Among them, four participants had lost their spouses and the average duration since their immigration was 34 years (see Table I).

**Table I:** Demographic characteristics of participants.

Name	Age	Gender	Length of stay (year)	Educational background	Marital status
Lee	77	Female	10	Bachelor	Widow
Park	73	Male	33	High School	Married
Jo	70	Female	33	High School	Married
Kwon	70	Female	45	High School	Married
Lee-Si	70	Male	25	High School	Married
Chen	66	Female	25	High School	Married
Kim	65	Male	32	PhD	Married
Lee-J	80	Male	52	PhD	Married
Choi	74	Male	46	Bachelor	Widow
Lee-W	65	Male	26	High School	Married
Kwon-F	65	Female	34	Bachelor	Widow
Jang	68	Male	43	PhD	Married
Jeun	82	Female	15	High School	Widow
Sujin	68	Female	31	High School	Married
Lee-F	85	Male	42	High School	Married
Kim-Q	68	Male	28	Bachelor	Married
Kim-S	76	Male	36	Bachelor	Married
Lee-F	75	Female	47	High School	Married

### Procedure for recruitment of participants

We contacted Korean communities in the northwestern United States to recruit study participants. The strategy for recruiting potential participants was that we put flyers on noticeboards at Korean community centers (e.g., churches, Korean markets, and Korean senior centers) and had meetings with the directors of Korean senior centers. After announcing this study information to the Korean community centers, five participants (i.e., Kwon: woman, aged 70; Lee-J: man, aged 80; Jang: man, aged 68; Lee-Si: man, aged 70; and Kim: man, aged 65) expressed their interest in participation. The investigator had meetings with them individually and delivered detailed information regarding the research purpose, confidentiality, consent forms, and study time frame. At the beginning of the data collection process, we gained confidentiality agreements through informed consent.

In the beginning, we encountered a challenge in recruiting participants and facilitating the process. Therefore, a snowball sampling strategy was employed according to Freeman, Palmer, and Baker (2006). As a result, we recruited additional 13 participants. According to the principles of theoretical saturation, Guest, Bunce, and Johnson (2006) had identified 12 participants with similar background characteristics, such as culture, race, ethnicity, and gender. When we interviewed our 17th participant, we discovered that no new data were emerging; the 18th participant had reached the saturation point. No payment was offered for participation in the study.

### Ethical considerations

We followed the procedure suggested by Miller (2000) to gain informed consent. We informed the participants that participation is voluntary and they can withdraw from the study at any time. In this study, only surnames (pseudonyms) were used to identify participants. The University Institutional Review Board approved these procedures (#39889).

### Data collection

We used semi-structured in-depth interviews to capture leisure behaviors of older Korean immigrants. Each interview lasted between 50 and 90 min. We developed a questionnaire to ascertain the leisure benefits associated with acculturation. We employed general and specific questions to capture specific aspects of participants’ experiences. Examples of the general questions were as follows: “Do you feel most comfortable speaking in English or Korean?”, “When did you move to the United States?”, “Could you tell me about your life story?”, and “With whom do you currently live?” To elicit rich emerging themes, the following specific open-ended questions were asked: “Why do you participate in the activities you mentioned?”, “What benefits do you experience when participating in these activities?”, and “What role, if any, has these activities had in helping you deal with the challenges in your life during your immigration process?” All participants expressed that they felt more comfortable with Korean when being interviewed. With the participants’ permission, all interviews were audio-recorded using an MP3 player and transcribed verbatim.

### Data analysis

We followed the six steps of data analysis presented by Smith et al. and Rafique and Hunt (2015). They were (a) reading and rereading, (b) initial noting, (c) developing emergent themes, (d) searching for connection among emergent themes, (e) moving to the next case, and (f) looking for themes across cases, which are briefly discussed here.

*Reading and rereading*: After producing raw data of each transcript, we read and reread them thoroughly, understanding and exploring participants’ experiences. This process allowed us to evaluate an overall sense of the entire interview process.

*Initial noting*. We conducted initial noting of each transcript. The initial notes were added to the left side of margin in the transcript. This step helped us to identify examples of different themes and understand participants’ lived experiences related to leisure behaviors.

*Developing emergent themes*: Based on the initial notes, we focused on developing themes by comparing and contrasting the patterns and connections between exploratory notes.

*Searching for connection among emergent themes*: With the emergent themes, we clustered connected themes in a meaningful way and created superordinate themes.

*Moving to the next case*: We scrutinized each transcript individually to explore new and unexpected themes, and this process was applied to all the cases. Each transcript contained rich quotes and narrative descriptions.

*Looking for themes across cases*: We constantly compared themes of each transcript with those of other cases. This process helped us to reconfigure and label themes and create clusters that collect each theme across cases.

### Trustworthiness in qualitative research

We employed strategies to increase the rigor in the data analysis (Kvale, 1996). Strategies were expert debriefing, back-translation, and member checking. With regard to member checking, we followed the guideline suggested by Peterson, Papes, and Eaton (2007) insofar as each participant was given an opportunity to rate his or her satisfaction with the interpretation and analysis of the data. Fifteen participants voluntarily participated in the member-checking process, and all found the summary of the themes and our interpretation of the data satisfactory.

Qualitative researchers stressed the importance of back-translation as a strategy for rigor in data analysis (Guillemin, Bombardier, & Beaton, 1993; Suh, Kagan, & Strumpf, 2009). Two bilingual research professionals, who were not associated with this research project, were invited to verify the translation from Korean to English. For this process, the research team randomly chose a 22-page set of translations and sent it to the two research professionals to undertake the back-translation. After 2 weeks, the research team had a conference call with them to validate the quality and conceptual equivalence of the translation. All agreed that there were no concerns regarding the conceptual meanings and content of the translation. In addition, each investigator has his or her expertise in qualitative research and demonstrated qualitative experience. This expert review process helps to increase the credibility of qualitative data by reducing interviewees’ personal biases.

## Results

Three themes emerged from participants’ personal statements and experiences: (a) experiencing psychological benefits, (b) strengthening social connections, and (c) coping with acculturative stress. The three themes highlight a variety of leisure activities that participants experienced, including volunteerism, culturally meaningful activities, home-based activities, and church-based activities as an effective strategy to deal with stress.

### Experiencing psychological benefits

This theme highlights the psychological benefits that seem to be related to engagement in leisure. Nearly all participants mentioned that they experienced psychological benefits including self-accomplishment, independence, and positive feelings. In addition, they shared that participation in leisure contributed to their sense of happiness and psychological comfort. For example, Choi (man, 74), who volunteered to teach judo to students, sought to deliver Korean culture, manners, and philosophy through judo, such as bowing to show respect to others. When he realized that his students had gained new cultural perspectives, he thought that it improved a sense of self-accomplishment.

Some participants believed that they gained positive feelings and emotions by using their leisure time in physical activities such as badminton. For example, Lee-W (man, 65) had suffered from alcohol issues, and playing badminton allowed him to find a new life opportunity that boosted his self-esteem and reduced unhealthy behaviors. He believed that through this badminton activity he developed friendships with other Korean immigrants and experienced fewer negative feelings about himself. He also said that his life had changed substantially because of his engagement in this activity.

Park (man, 73), who engaged in activities such as raising animals and practicing yoga, experienced psychological benefits, such as an increased sense of comfort and happiness. When he raised these animals, he found peace and happiness. In addition, his participation in these activities enabled his grandchildren to enjoy themselves via new pursuits, such as horseback riding and swimming, which in turn made him feel satisfied and fulfilled. He expressed more happiness when he was watching his grandchildren ride horses or swim, rather than when he engaged in his own favorite leisure pursuits.

The participants shared about their adaptation difficulties in areas such as transportation. For example, Jeun (woman, 82) made an effort to independently use public transport and pursued new, personally meaningful activities, including riding the bus and visiting new places. After a while, she gained information about the bus routes and had developed real expertise in this area. One of her leisure activities was to take a trip to the city center and local areas by taking the bus herself. As a result, she said that she developed a sense of independence and experienced enhanced self-confidence, which in turn resulted in a sense of accomplishment and happiness.

Volunteering was one of the activities that provided psychological benefits to participants. They were most likely to receive psychological and mental health benefits through volunteer activities, such as helping others (i.e., older Korean immigrants and the underprivileged). For example, Kwon (woman, 70) mentioned that she provided transportation for older Korean immigrants who could not drive. Through this activity, she mentioned that she derived psychological and emotional benefits from *people*, not from her actions.

Sujin (woman, 68) shared a similar experience related to psychological benefits as a result of volunteering activities. In her case, she organized a social activity for older Korean immigrants who felt depressed and lonely. Helping others tends to be linked with some demands such as time, energy, and age-related physical challenges, but engaging in this activity was emotionally rewarding. As a result, she experienced a sense of happiness.

These life examples show how leisure contributes to psychological benefits, such as happiness, self-confidence, accomplishment, and independence, among older Korean participants. It seems that personally meaningful activities play an essential role in their lives as it serves as an important means of enhancing positive feelings, self-accomplishment, and a sense of independence.

### Strengthening social connections

This theme describes that leisure helps participants to gain the social benefits related to strengthening social connections with others. According to the participants, leisure provided them with an opportunity to alternate ways of socializing with others and helped them adapt to new societies using each other as support. As Lee-W (man, 65) said,

… During our conversations, we exchanged useful information related to immigration life and share our stories. For example, if someone needed a plumber, based on our experience, we recommended a good plumber who was reliable and had good skills. These activities help us to adapt to new things.

Based on his experience, leisure activities served as a method of providing social support to each other and aiding in cultural adaptation. This may mean that immigrants needed practical information about how to adapt to a new place. Regarding Lee-W's (man, 65) experience, it seems that leisure provided an environment in which immigrants shared practical information related to acculturation, which resulted in social interactions.

Similar to Lee-W's experience, Jang (man, 68) enjoyed playing golf with his retired Korean friends. He mentioned that after their golf game, they had lunch together as a group and discussed various topics, such as politics, sports, and the economy. Through playing golf, his friendships were established and maintained, or, as he put it, “This is a good social support group.”

Some individuals participated in traditional Korean games, such as *yut-nori*, *go-stop*, and *baduk*. As a result of their participation in these activities, they developed a sense of friendship and expanded their social networks. For example, Jeun (woman, 82) stated,

Sometimes, we played traditional Korean games, such as *yut-nori*, *go-stop*, et cetera. Playing such Korean games together helps us develop our friendships and relieves any stress related to loneliness … Many older adults living in Greensboro are very lonely. There are few communities or nursing homes for older Koreans. Because of loneliness, we hang out together and gather to play some Korean games.

According to the participants, church was a unique place in which older Korean immigrants were able to socialize with others as part of church service. In addition, church was described as a facilitator that provided opportunities for interaction among older Korean immigrants and other people who had similar experiences. Jo (woman, 70) mentioned that church was a good place to socialize, and she was eager to engage in various church-based activities such as Korean food festival and Korean language event. She commented that church was an important place for her to relieve her loneliness because she made many friends there.

Lee-F (man, 85) started a social gathering in which he shared food with his Korean friends at his home. By sharing food that he harvested, he mentioned that he had the opportunity to interact with other Korean immigrants. In addition, some of his friends also gardened and exchanged information related to gardening. It seems that the leisure-time environment provides an opportunity to build on Korean culture and improve social relationships with others.

Based on the participants’ experiences, leisure plays an important part in their lives, as it provides an opportunity for them to have meaningful interpersonal interactions with others. Older Korean immigrants used leisure as a means of providing opportunities for socialization with others and developing emotional and social support. Leisure enabled participants to share similar experiences and develop deeper, more intimate friendships. It seems that leisure is an important pastime through which participants obtained social benefits, which led to a reduction in stress and feelings of loneliness.

### Coping with acculturative stress

This theme contextualizes the participants’ experiences that seemed to be related to coping with acculturative stress. The participants had shared that they experienced various stressors related to acculturation, such as adaptation difficulties, language barriers, family issues, children's struggles, and racial conflicts and tension right after their immigration. To cope with the adaptation challenges, participants had to be engaged in various types of leisure activities. They mentioned that leisure helped them to cope with adaptation challenges and reduce the level of acculturative stress.

Demands related to cultural role were reported by four female participants because they had to embrace different cultural roles, including now being expected to take care of household chores. The participants shared that the household chores were the responsibility of their daughters-in-law when they lived in Korea. They said that a different cultural role was a stressful event. Therefore, they expressed that both social activities and spiritual activities helped them to deal with the acculturative stress related to different cultural roles in their lives. For example, Lee (woman, 77) stated,

Since I developed my faith through interactions with other older adults and exchanged our life experiences together; I began to find some peace and comfort. After that, my relationship with my daughter-in-law and granddaughter improved and we have a better understanding of each other.

She believed that having faith and sharing her life with others became a better way of dealing with such stress.

Through these social gatherings, Jo (woman, 70) mentioned that she had developed a deep sense of faith and shared her life stories, regarding her relationship with her daughter-in-law, with other Korean immigrants so that she could develop a better understanding of the new cultural roles in her home. Similarly, she participated in gardening with her friends, and it seems that the social support formed through this social activity was helpful in developing coping mechanisms.

Participation in community-based activities and spiritual activities was reported as facilitators that enabled participants to deal with acculturative stress. This is evident from the experiences of Kim-Q (man, 68), who is a faculty member in a university. Kim mentioned that he had experienced challenges associated with acculturation, such as language barriers, cultural and ethnic differences, and racial discrimination at his institution. Therefore, it appeared that the most effective way of coping with acculturative stress was to engage in spiritual activities such as Bible study and missionary trips. He stated, “My spiritual activities helped me to deal with a great deal of stress.” Kim's coping strategies seemed to be similar to Chen's (woman, 66) experiences. Chen used various church-based activities such as Bible study and Korean festival to develop her faith and deal with acculturative stress.

In this study, the participants reported that they tended to engage in physical activities during their leisure time in order to cope with acculturative stress. Badminton was one of the physical activities that Lee-W (man, 65) engaged in to reduce his unhealthy behaviors like drinking problems, and it assisted him to deal with acculturative stressors in a healthier way. Lee mentioned that engagement in the badminton tended to provide opportunities to socialize with other older Korean immigrants while sharing food and expanding their social networks. This may mean that gathering enabled Lee and other older Korean immigrants to help each other to cope with acculturative stress.

The participants’ life experiences seemed to illustrate that engaging in leisure serves as a coping mechanism to deal with acculturative stress. Similarly, the participants’ experiences provided an insight into and understanding of the importance of leisure in older Korean immigrants. In particular, older Korean immigrants used leisure as an effective strategy to deal with different cultural roles, expand their social networks, and develop social support groups.

## Discussion

This study sought to explore the leisure benefits associated with acculturation among older Korean immigrants. Overall, the findings of this study indicate that engagement in leisure seems to provide older Korean immigrants with psychological, emotional, and social benefits. Likewise, the benefits of leisure appeared as an effective coping mechanism that assisted older Korean immigrants to deal with acculturative stress. This study suggests that leisure can lead to substantial health and well-being.

Leisure scholars demonstrate that leisure contributes to substantial health benefits, such as physical, psychological, cognitive, and social benefits, among older adults (Kim, Yamada, Heo, and Han, 2014; Kim & Kim, 2013; Mannell, 2007). The findings of this study are aligned with that of previous studies in that through leisure participation older Korean immigrants obtained health benefits, such as psychological well-being and social benefits. Furthermore, this study suggests that by participating in leisure older Korean immigrants attained positive affect, enhanced a sense of independence, and received an emotional and social support from others. These benefits can serve as facilitators of quality of life and successful aging.

Previous studies have demonstrated that older adults used leisure as a way to cope with and adapt to conditions and transitions (Hutchinson, Bland, & Kleiber, 2008; Nimrod, 2009). They mainly focused on the lives of older adults, mainly those who are white, middle class, and non-Hispanic. The findings of the current study expand the body of knowledge that leisure helped older Korean immigrants to develop the ability to cope with acculturative stress and adaptation difficulties. By participating in leisure, older Korean immigrants reduced their acculturative stress, facilitated cultural understanding, and developed social support groups.

Several researchers (Nimrod, 2009; Son, Kerstetter, Yarnal, & Baker, 2007) found that leisure provided rich opportunities for older adults to share leisure resources and practical information (e.g., recreation, shopping, and travel). The current study supports the idea that leisure is used as a means of sharing practical information related to acculturation among older Korean immigrants. Furthermore, the findings of this study are consistent with that of previous studies that show that leisure provides older adults with opportunities to develop their perceptions of belonging, acceptance, and support (Hutchinson, Bland, & Kleiber, 2008; Son et al., 2007). This study suggests that older Korean immigrants developed a sense of acceptance and connectedness with each other through leisure.

According to Kim, Heo, & Kim, 2014 study, Korean immigrants organized their own sports club in which the members had the same ethnic identities and, as a result, they experienced psychological well-being and created their unique cultural world in a context of sports. The finding of this study is consistent with this idea that older Korean immigrants gained various social, cultural, and psychological benefits by engaging in leisure activities with other Korean immigrants. It suggests that by interacting with the same ethnic group, older Korean immigrants maintain their cultural identities through leisure activities that have created a psychological comfort zone.

## Limitations and future research

There are some limitations that need to be addressed. First, this study mainly focused on how leisure leads to health benefits among older Korean immigrants. There can be multiple factors that influence the leisure behaviors of older Korean immigrants, such as gender, the level of acculturation, educational background, immigration motivation, and/or length of stay. Future research is needed to examine what factors positively influence leisure behaviors among older Korean immigrants.

This study did not measure the level of acculturative stress. Older Korean immigrants may report a different level of acculturative stress and adaptation challenges. It would be helpful if future researchers examine how leisure is related to levels of acculturative stress and adaptation challenges.

The last limitation is with regard to a limited population. This study examined the leisure behaviors among older Korean immigrants only. Other older ethnic immigrants can pursue different types of leisure, and their own cultures can influence their leisure behaviors. Future research is needed to examine how other older immigrants experience their leisure behaviors and their benefits and constraints.

## Conclusion

This study has advanced the literature on leisure by acknowledging the leisure benefits of older Korean immigrants. The findings provide an important contribution to the literature that leisure plays a vital role in improving psychological and social benefits. In particular, leisure participation allows older Korean immigrants to create an emotional and social support system and deal with acculturative stress and adaptation challenges. This study suggests that leisure participation contributes to successful aging among older Korean immigrants.
